# Supplementary material for: Vastly extended drug release from poly(pro-17β-estradiol) materials facilitates in vitro neurotrophism and neuroprotection
Source: Nat Commun. 2019 Oct 23;10:4830. doi: 10.1038/s41467-019-12835-w (PMC6811552; doi:10.1038/s41467-019-12835-w)
Supplement: Supplementary file 1 — Supplementary Information [file 41467_2019_12835_MOESM1_ESM.pdf]

Supplementary Information for:

D'Amato *et al*.

Title: "Vastly Extended Drug Release from Poly(pro-17β-Estradiol) Fibers and Films Enables in vitro Neuroprotection, Neurotrophism, and Neuron Contact Guidance"

Supplementary Information

Vastly Extended Drug Release from Poly(pro-17β-Estradiol) Fibers and Films Enables in vitro Neuroprotection, Neurotrophism, and Neuron Contact Guidance.

Anthony R. D'Amato, Devan L. Puhl, Samuel A. T. Ellman, Bailey Balouch[1], Ryan J. Gilbert, Edmund F. Palermo**

Supplementary Methods

Materials: Poly-L-lactic acid (PLLA, NatureWorks; grade 6201D, Lot #9051-89-2) was purchased from Cargill Dow LLC (Minnetonka, MN). All chemical reagents and organic solvents used for chemical synthesis, characterization, and electrospinning were purchased from Sigma. These include chloroform (product no. Cx1056), dichloromethane (DCM, product no. 270997), tetrahydrofuran (THF, product no. TX0277), pyridine (product no. 270970), deuterated chloroform (product no. 416754), E2 (product no. E2768), Deuterated MeOH (MeOH-d4, product no. 151947), hydrogen peroxide (H_2O_2, product no. H1009), and 2,2-dimethoxy-2-phenylacetophenone (DMPA, product no. 196118). 15x15 mm square glass coverslips used in film casting and electrospinning were purchased from Knittel Glass (Brausenweig, Germany).

Instrumentation: Proton and carbon nuclear magnetic resonance (^{1}H and ^{13}C NMR) spectra were recorded using 500 MHz Agilent NMR spectrometer at 25 °C. NMR chemical shifts were reported in parts per million (ppm, δ) and referenced to tetramethylsilane ($(CH_3)_4Si$, 0.00 ppm). Residual solvent signals for ^{1}H NMR: $CDCl_3$ (δ 7.26), DMSO-d6 (δ 2.50) and ^{13}C NMR: $CDCl_3$ (δ 77.0), DMSO-d6 (δ 39.5). Gel permeation chromatography (GPC) performed on an Agilent Technologies 1260 Infinity GPC with THF as the mobile phase (100 µL injection volume, 1 mL/min flow rate). P1 MW was then determined using monodisperse polystyrene standards. High resolution mass spectroscopy (HRMS) was performed on Thermo LTQ Orbitrap XL instrument at resolution 30,000 (at m/z 400) and mass accuracy better than 3 ppm. Samples were injected in the ESI source using Agilent 1200 HPLC system in methanol as a mobile phase at flow rate 50 µL/min.

Synthesis of 1: In a nitrogen-filled glovebox, 17β estradiol (5.00 g, 18.4 mmol, 1 eq.) was dissolved in anhydrous THF (50 mL) in a 250 mL Schlenk flask with a magnetic stir bar, and anhydrous pyridine (14.8 mL, 0.184 mol, 10 eq.) was added. Allyl chloroformate (11.7 mL, 0.110 mol, 6 eq.) was dissolved in anhydrous THF (12 mL) in a 50 mL addition funnel that was secured to the Schlenk flask and capped with a rubber septum. The apparatus was removed to an inert gas Schlenk line and the flask was cooled to 0 °C over an ice bath with stirring for ~ 15 min. Under N_2, the allyl chloroformate solution was added dropwise to the cooled solution in the flask. After complete addition, the flask was removed from the ice bath and allowed to warm to rt overnight with stirring. The resulting mixture was diluted with ethyl acetate (~75 mL) in a 250 mL separatory funnel, extracted sequentially with 0.1 M HCl aq. (100 mL), deionized water (100 mL), 0.1 M NaOH aq. (100 mL), deionized water (100 mL), and brine (100 mL). The organic phase was dried over anhydrous sodium sulfate, filtered, and concentrated under reduced pressure. The crude oil was then purified by silica gel column chromatography (ethyl acetate: hexanes = 2:3 v/v, R_f ~ 0.7) to give a viscous yellow oil that was dried *in vacuo* overnight (3.3524 g, 41.45 % yield). 1: ^{1}H NMR (500 MHz, DMSO-d6): δ = 7.28 (d, J = 9.0 Hz, 1H), 6.93 (d, J = 8.5 Hz, 1H), 6.89 (s, 1H), 5.99-5.90 (m, 2H), 5.39-5.22 (m, 4H), 4.69 (d, J = 5.5 Hz, 2H), 4.58 (d, J = 8.5 Hz, 2H), 4.53 (t, J = 8.5 Hz, 1H), 2.79 (br, 2H), 2.30-2.28 (m, 1H), 2.22-2.13 (m, 2H), 1.80 (m, 2H), 1.67 (m, 1H), 1.54 (m,

1H), 1.42-1.28 (br m, 6H), 0.77 (s, 3H). ^{13}C NMR (500 MHz, DMSO-d6): δ = 154.30, 153.01, 148.54, 137.90, 137.64, 132.31, 131.83, 126.41, 120.91, 118.77, 118.33, 118.25, 85.63, 68.59, 67.69, 48.80, 43.31, 42.47, 37.68, 36.29, 28.88, 27.02, 26.41, 25.60, 22.63, 11.78. HRMS (ESI) [M+H$^+$] calcd: 441.2272, found: 441.2279.

Synthesis of P1: The pro-E2 monomer 1 (3.3524 g, 7.61 mmol, 1.00 eq) and 2,2'-(ethylenedioxy)diethanethiol (1.3998 g, 7.61 mmol, 1.00 eq) were added to a 20 mL glass scintillation vial containing a magnetic stir bar. The UV photoinitiator 2,2-Dimethoxy-2-phenylacetophenone (DMPA, 0.0195 g, 0.076 mmol, 0.01 eq) was dissolved in THF (10 mL) and added to the monomer solution via pipette. The solution was concentrated under reduced pressure to remove all solvent, deoxygenated by bubbling with N_2 for 10 min, and stirred overnight at rt under a 4-W handheld UV lamp at a distance of 2 cm. Then, the resulting solidified mixture was dissolved in DCM and precipitated into cold MeOH. The precipitate was concentrated by centrifugation (4500 rpm, 15 min), the supernatant liquid was decanted, and the pellet was dried *in vacuo* overnight to obtain an off-white gummy substance (3.6467 g, 84 % yield). P1: ^{1}H NMR (500 MHz, $CDCl_3$): δ = 7.28 (d, 1H overlapping solvent), 6.92 (d, J = 8.5 Hz, 1H), 6.89 (s, 1H), 4.60 (t, J = 8 Hz, 1H), 4.33 (t, J = 6.2 Hz, 2H), 4.22 (t, J = 6.1 Hz, 2H), 3.76 (m, 1H), 3.68-3.63 (br m, 8H), 2.86 (m, 2H), 2.75-2.63 (m, 8H), 2.31-2.23 (m, 3H), 2.02-1.25 (m, 18H), 0.84 (s, 3H). ^{13}C NMR (500 MHz, DMSO-d6): δ = 151.16, 153.84, 148.76, 138.18, 137.95, 126.42, 120.91, 118.01, 86.20, 71.09, 70.92, 70.27, 67.06, 66.40, 66.25, 49.60, 43.86, 42.88, 38.06, 36.74, 31.36, 29.44, 28.75, 28.70, 28.65, 27.35, 26.88, 25.93, 23.04, 11.92 Gel permeation chromatography (GPC, PS standards, THF, 25 °C): M_n = 84 kg/mol, Đ = 3.73.

Dissociated DRG Purity Assessment: DRG were harvested and dissociated according to previously established methods[1]. First, whole DRG were submerged in a buffer containing 0.1% Trypsin (Corning) and 1 mg/mL collagenase A (Sigma) in PBS and placed in a cell culture incubator for 50 min. The tubes were inverted every 10 min to resuspend the DRG and promote enzymatic dissociation of the tissues. Following the 50 min incubation, DRG were centrifuged at 300 g for 5 min and the supernatant was removed. The DRG were then submerged in a buffer containing 0.1% Trypsin in PBS for 10 min. Following the 10 min incubation, serum-containing media was added to neutralize trypsinization. The cells were gently triturated and then centrifuged at the previously stated parameters. The supernatant was removed, and cells were brought up in neuron media containing neurobasal media (Gibco) supplemented with 50 ng/mL nerve growth factor (sigma), 1% Pen/Strep (Gibco), 0.5mM L-Glutamine (Gibco), and 1% B27 media supplement (Gibco). To assess the purity of neuron cultures, dissociated DRG were seeded onto glass coverslips in triplicate. Coverslips were coated with a 500 ng/mL solution of laminin (Invitrogen) for 1 hr prior to seeding to promote cell adhesion. Dissociated DRG were cultured, immunostained and imaged according to the protocol described in the main text. After paraformaldehyde fixation, a 1:500 dilution of S100 primary antibody (Dako) and a 1:1000 dilution of AlexaFluor goat-anti-rabbit 594 secondary antibody (Invitrogen) were added in conjunction with RT97 primary antibody against neurofilament and AlexaFluor donkey-anti-mouse 488 secondary antibody, respectively. S100 is expressed by Schwann cells and neuron subpopulations, whereas neurofilament is only expressed in neurons (Supplementary Fig. 12).

The percentage of neurons resulting from DRG dissociation was determined by counting the number of neurofilament-positive cells and dividing by the total number of cells in each image. This analysis was conducted in triplicate with three separate batches of dissociated DRG. Dissociated DRG cultures contained approximately 16% neurons and 84% Schwann cells.

Supplementary Figures:

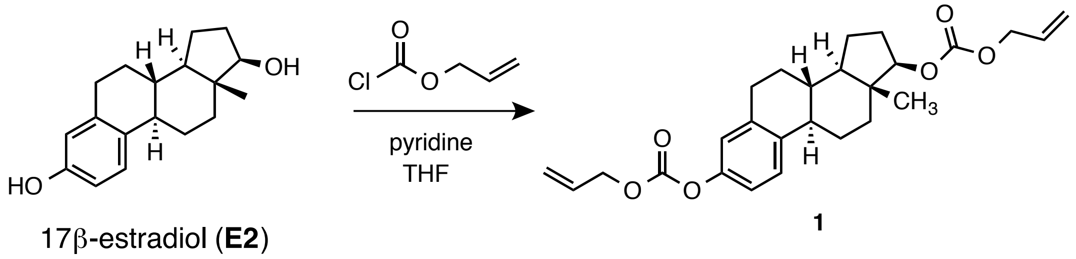

Supplementary Fig. 1 Synthesis of the diallylcarbonate of 17β estradiol (1).

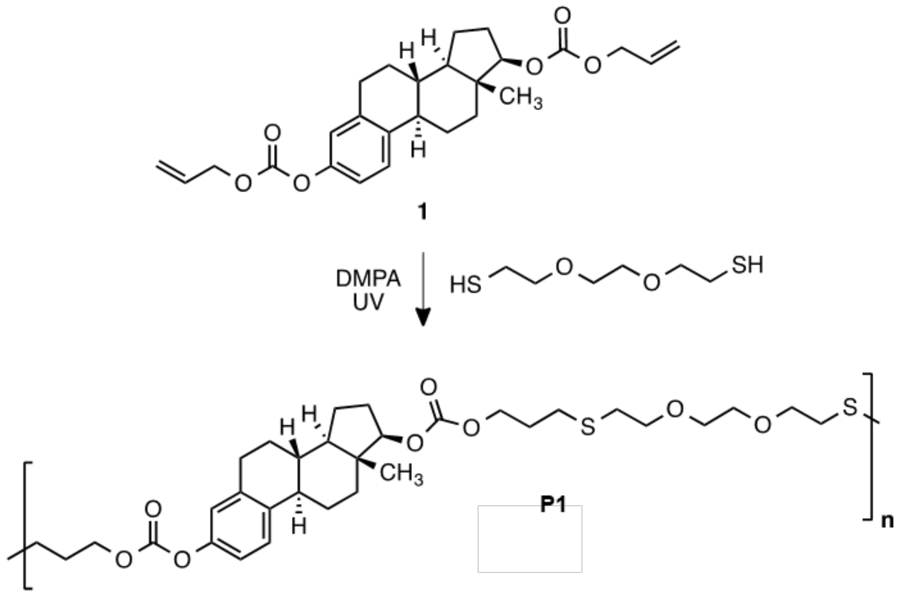

Supplementary Fig. 2 Polymerization of 1 and dithiol to yield P1.

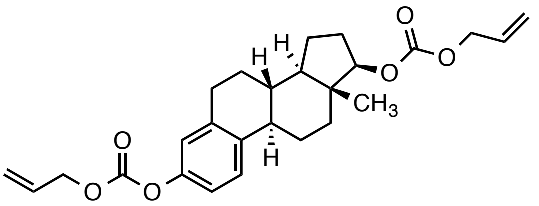

1

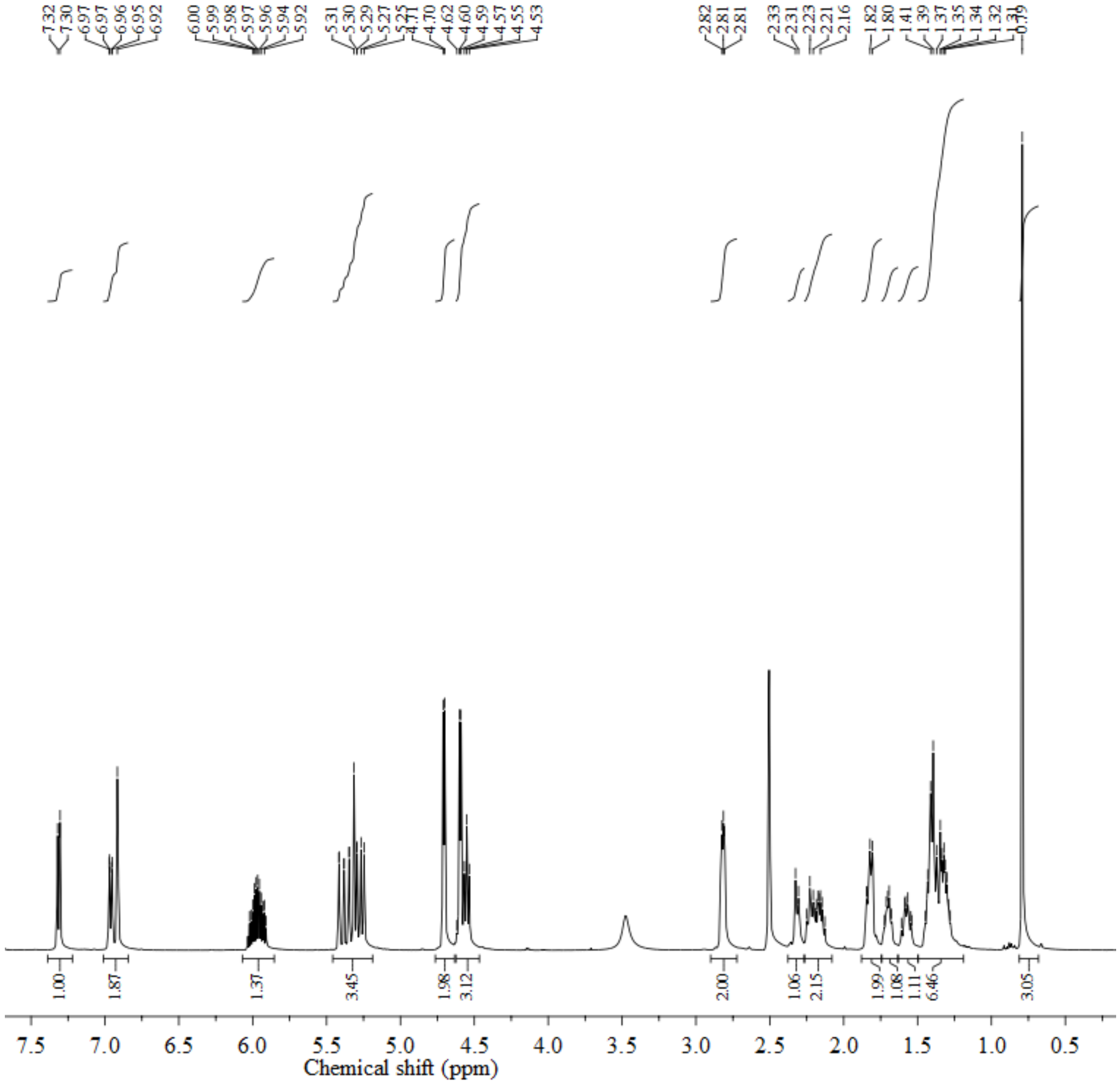

Supplementary Fig. 3 ^{1}H NMR spectrum of 1 in DMSO-d6.

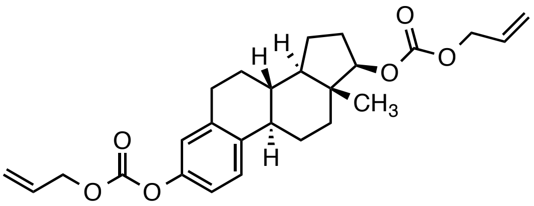

1

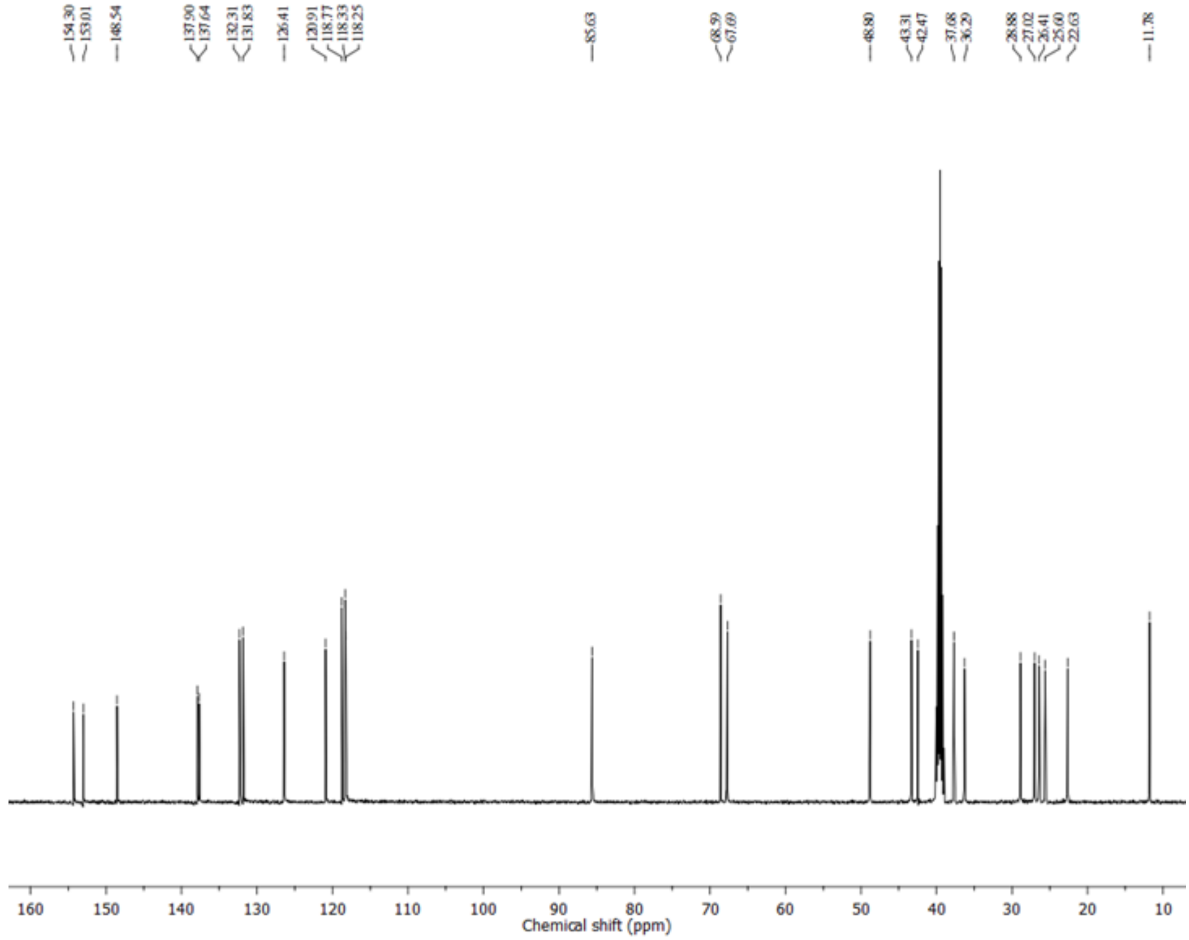

Supplementary Fig. 4 ^{13}C NMR spectrum of 1 in DMSO-d6.

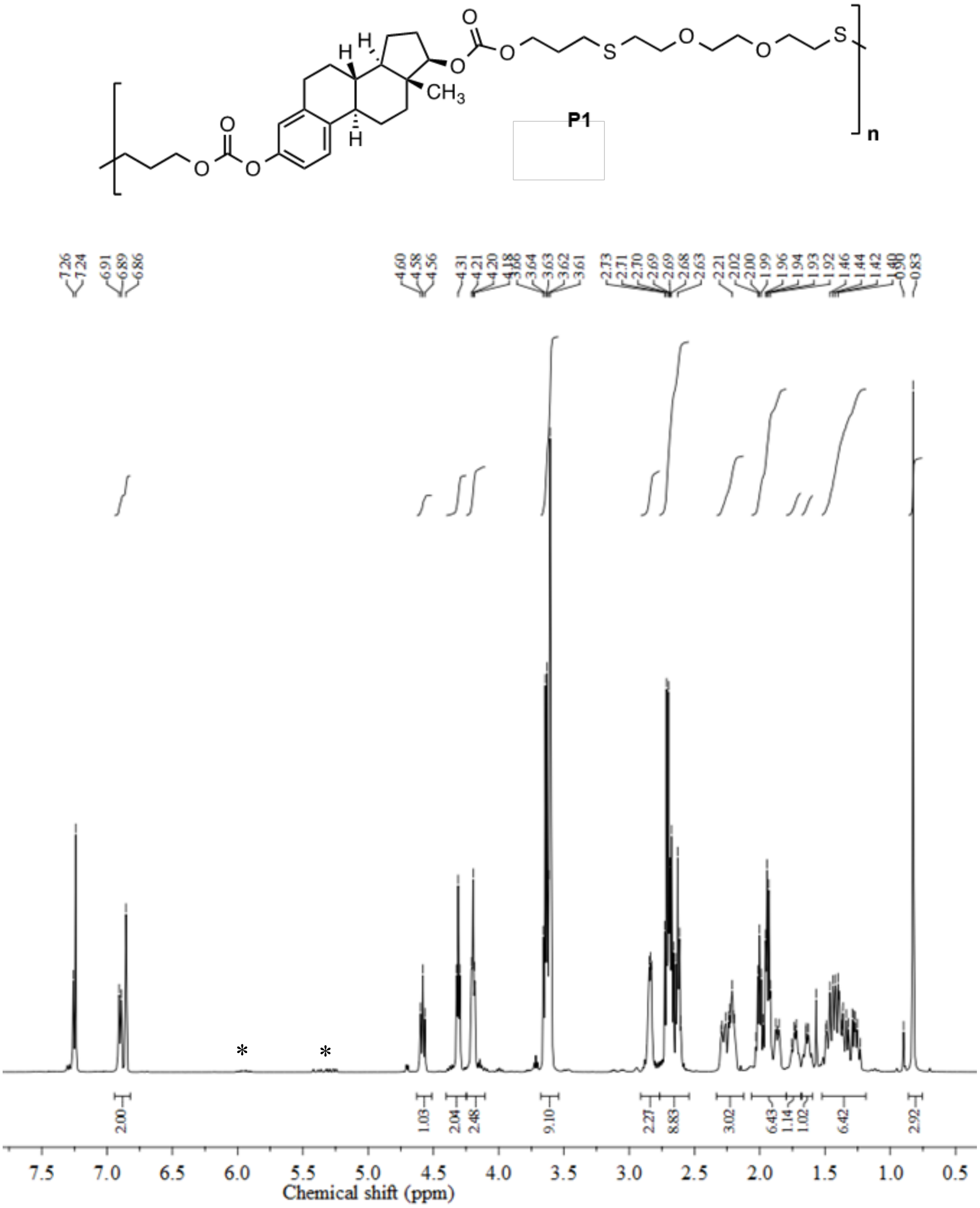

Supplementary Fig. 5 ^{1}H NMR spectrum of P1 in $CDCl_3$. The * symbols denote the faint presence of unreacted allyl groups on the polymer chain ends.

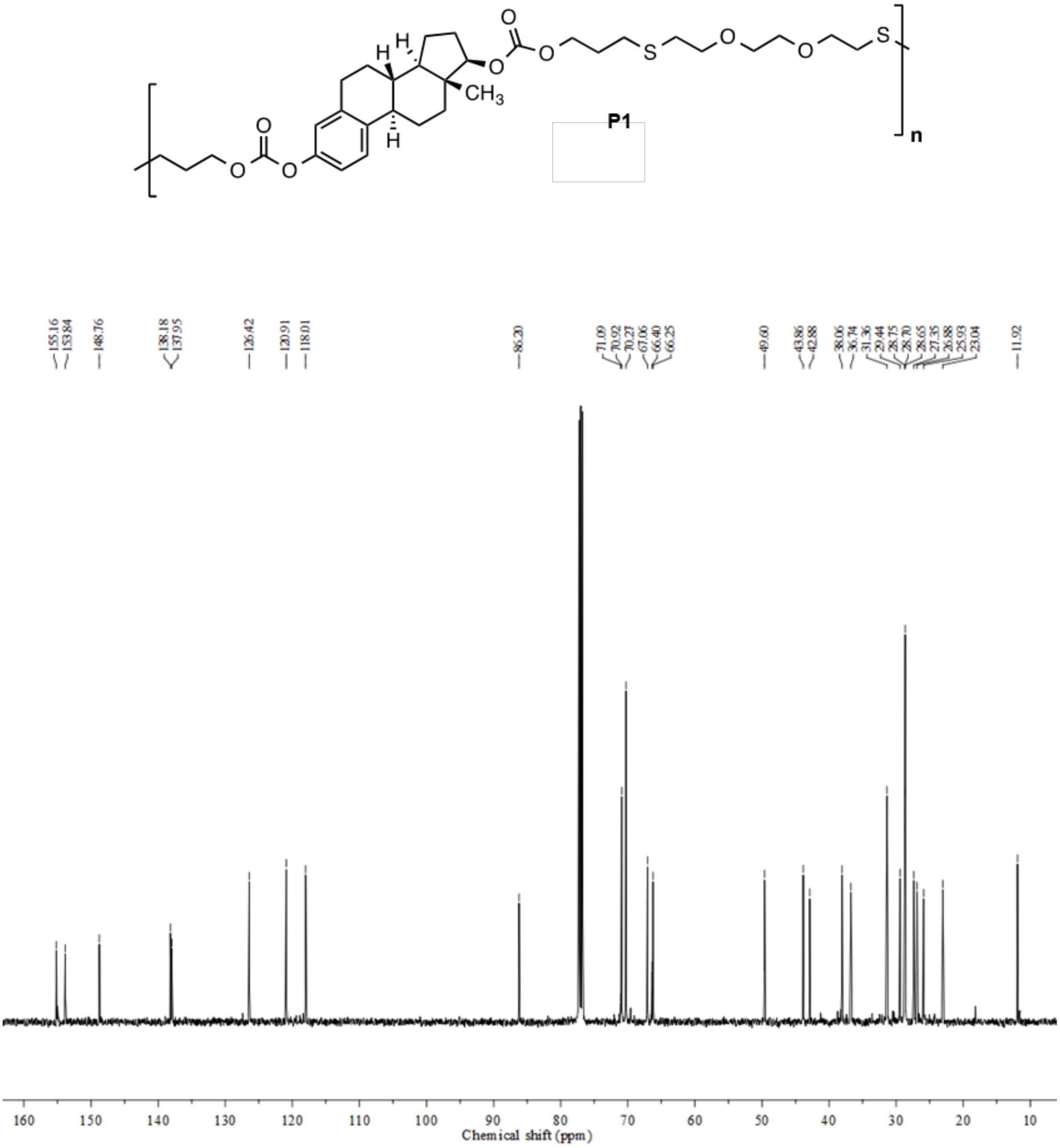

Supplementary Fig. 6 ^{13}C NMR spectrum of P1 in $CDCl_3$.

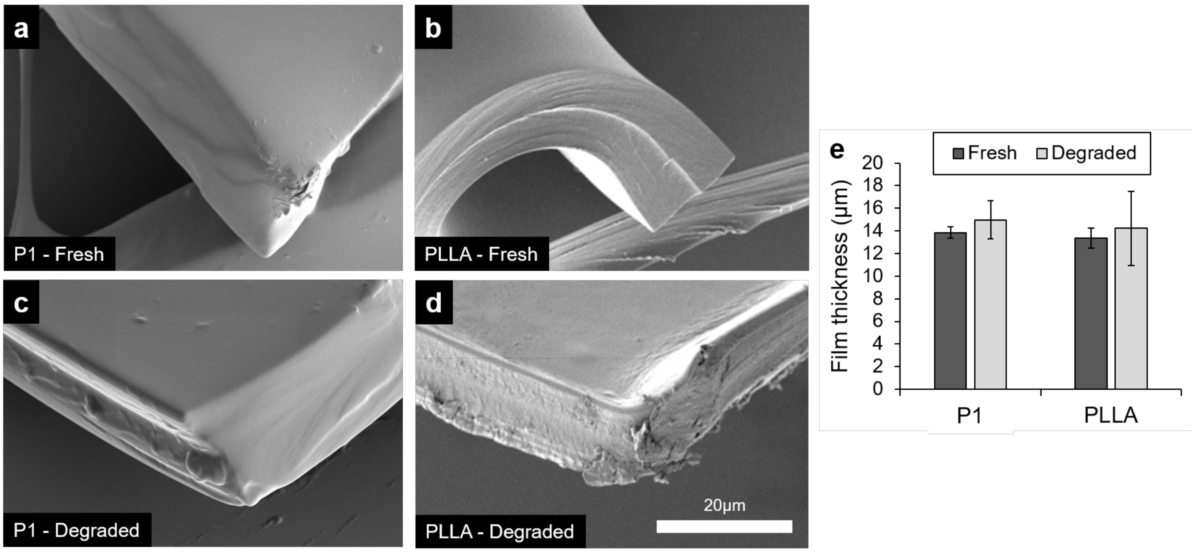

Supplementary Fig. 7 P1 and PLLA film degradation mechanism in neuron media at 37 °C for 5 days. **a-d** SEM of P1 and PLLA films before and after degradation. **c** P1 and PLLA film thickness before and after the 5-day incubation. (n=3, all values are mean ± std. dev., SEM scale bar is 20 μm)

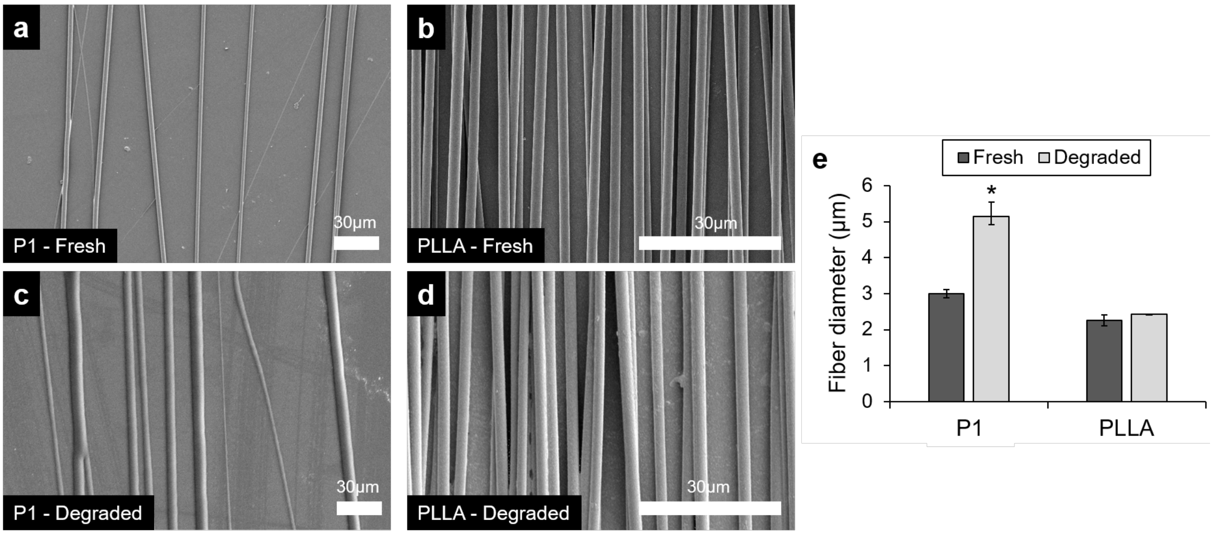

Supplementary Fig. 8 P1 and PLLA fiber degradation mechanism in neuron media at 37 °C for 5 days. **a-d** SEM of P1 and PLLA fibers before and after degradation. **c** P1 and PLLA fiber diameter before and after the 5-day incubation. (n=3, all values are mean ± std. dev., *p<0.05 compared to fresh P1 fiber control via Tukey's HSD, SEM scale bars are 30 μm)

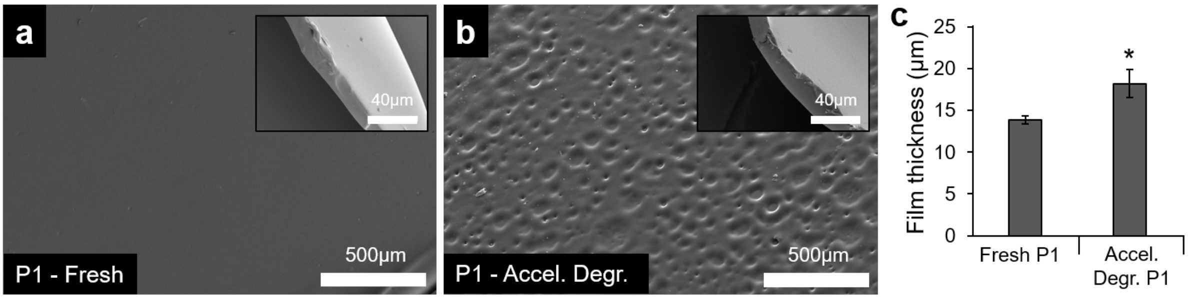

Supplementary Fig. 9 Accelerated P1 film degradation mechanism in water at 80 °C. **a-b** SEM of P1 films before and after accelerated degradation with inlaid SEM images of film cross sections. After accelerated degradation, P1 films exhibited surface pitting, and **c** a significant increase in cross-sectional film thickness. (n=3, all values are mean ± std. dev.,

*p<0.05 compared to fresh P1 film control via Tukey's HSD, SEM scale bars are 500 µm in large images, and 40 µm in cross-section inlays)

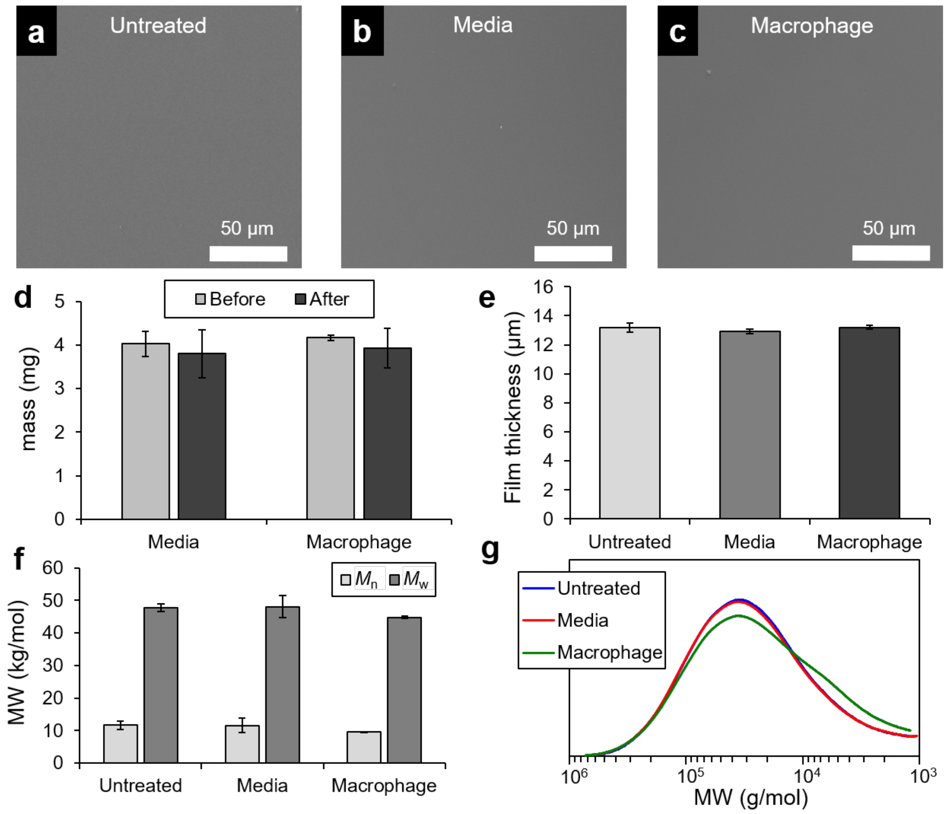

Supplementary Fig. 10 P1 film macrophage-mediated erosion. SEM of P1 films **a** untreated, **b** submerged in media, or **c** exposed to activated macrophages for 7 days. **d** P1 film mass before and after 7-day incubation in media or with activated macrophages. **e** P1 film thickness, **f** P1 number average (M_n) and weight average (M_w) molecular weight, and **g** P1 molecular weight distribution of untreated films and films incubated in media or with activated macrophages for 7 days. (n=3, all values are mean ± std. dev., SEM scale bars are 50µm)

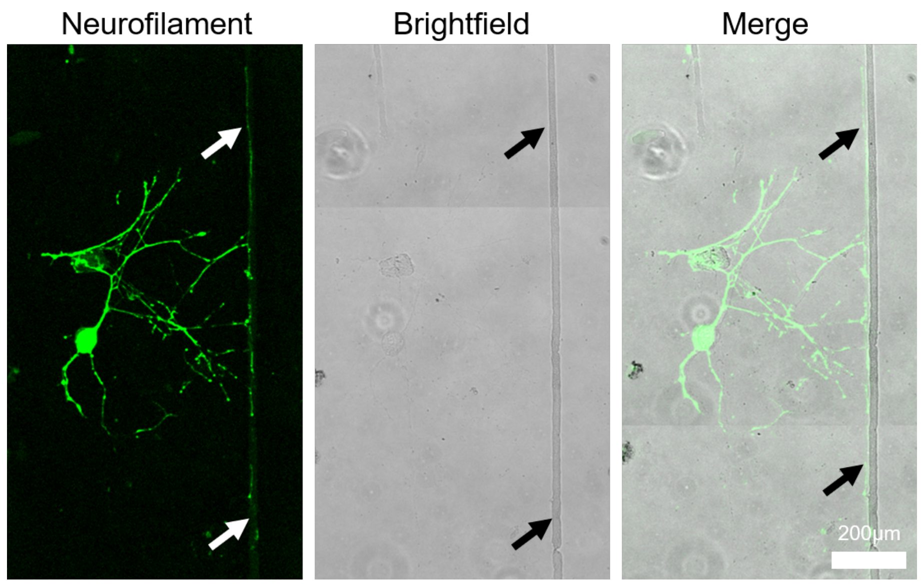

Supplementary Fig. 11 Neurons that adhered to glass coverslips and extended neurites that either did or did not encounter a P1 fiber. (Arrows highlight P1 fiber location and/or neurites growing along a P1 fiber, scale bar is 200 µm)

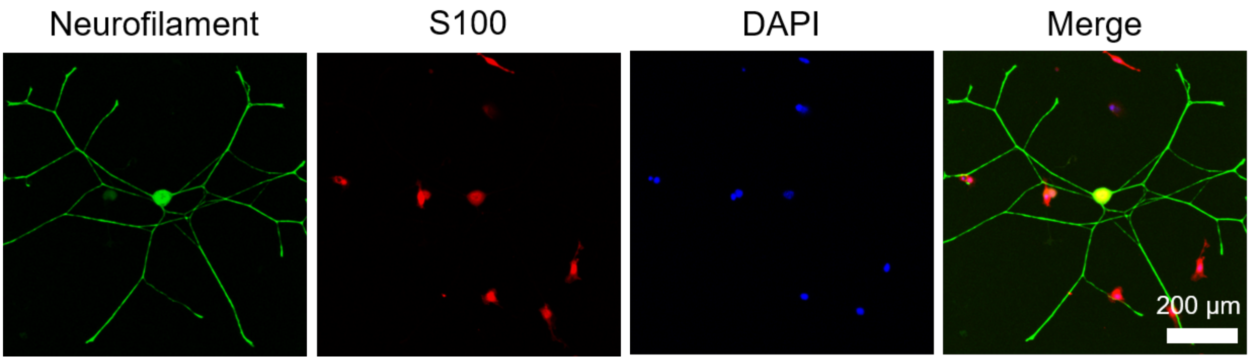

Supplementary Fig. 12 Neurofilament and S100 immunofluorescent images used to determine neuron purity in dissociated DRG cultures. (scale bar is 200 µm).

Reference

1. D'Amato, A. R. *et al.* Exploring the effects of electrospun fiber surface nanotopography on neurite outgrowth and branching in neuron cultures. *PLOS ONE* **14**, e0211731 (2019).
