# Supplementary material for: Vastly extended drug release from poly(pro-17β-estradiol) materials facilitates in vitro neurotrophism and neuroprotection
Source: Nat Commun. 2019 Oct 23;10:4830. doi: 10.1038/s41467-019-12835-w (PMC6811552; doi:10.1038/s41467-019-12835-w)
Supplement: Supplementary file 3 — Reporting Summary [file 41467_2019_12835_MOESM3_ESM.pdf]

nature research

Corresponding author(s): Edmund F. Palermo
Ryan J. Gilbert

Last updated by author(s): Sep 27, 2019

Reporting Summary

Nature Research wishes to improve the reproducibility of the work that we publish. This form provides structure for consistency and transparency in reporting. For further information on Nature Research policies, see Authors & Referees and the Editorial Policy Checklist.

Statistics

For all statistical analyses, confirm that the following items are present in the figure legend, table legend, main text, or Methods section.

n/a	Confirmed	
☐	☒	The exact sample size (*n*) for each experimental group/condition, given as a discrete number and unit of measurement
☐	☒	A statement on whether measurements were taken from distinct samples or whether the same sample was measured repeatedly
☐	☒	The statistical test(s) used AND whether they are one- or two-sided<br>*Only common tests should be described solely by name; describe more complex techniques in the Methods section.*
☒	☐	A description of all covariates tested
☒	☐	A description of any assumptions or corrections, such as tests of normality and adjustment for multiple comparisons
☐	☒	A full description of the statistical parameters including central tendency (e.g. means) or other basic estimates (e.g. regression coefficient) AND variation (e.g. standard deviation) or associated estimates of uncertainty (e.g. confidence intervals)
☐	☒	For null hypothesis testing, the test statistic (e.g. *F*, *t*, *r*) with confidence intervals, effect sizes, degrees of freedom and *P* value noted<br>*Give P values as exact values whenever suitable.*
☒	☐	For Bayesian analysis, information on the choice of priors and Markov chain Monte Carlo settings
☒	☐	For hierarchical and complex designs, identification of the appropriate level for tests and full reporting of outcomes
☒	☐	Estimates of effect sizes (e.g. Cohen's *d*, Pearson's *r*), indicating how they were calculated

Our web collection on statistics for biologists contains articles on many of the points above.

Software and code

Policy information about availability of computer code

Data collection	No software was used for data collection.
Data analysis	Data analysis was conducted using JMP IN Software (Release 8.0.1).

For manuscripts utilizing custom algorithms or software that are central to the research but not yet described in published literature, software must be made available to editors/reviewers. We strongly encourage code deposition in a community repository (e.g. GitHub). See the Nature Research guidelines for submitting code & software for further information.

Data

Policy information about availability of data

All manuscripts must include a data availability statement. This statement should provide the following information, where applicable:

- Accession codes, unique identifiers, or web links for publicly available datasets
- A list of figures that have associated raw data
- A description of any restrictions on data availability

All data supporting the findings in the manuscript are included in the main manuscript and/or supplemental information document. Supporting raw data will be made available upon reasonable request from the corresponding authors. Data also at FigShare, https://doi.org/10.6084/m9.figshare.c.4681220.v1

Field-specific reporting

Please select the one below that is the best fit for your research. If you are not sure, read the appropriate sections before making your selection.

☒ Life sciences ☐ Behavioural & social sciences ☐ Ecological, evolutionary & environmental sciences

Life sciences study design

All studies must disclose on these points even when the disclosure is negative.

Sample size	Sample sizes were chosen based on previous studies using similar cell culture models and drug release characterization techniques.
Data exclusions	No data were excluded from the analyses.
Replication	All experiments were able to replicated in this study. Each cell culture condition was studied with multiple replicates, all of which exhibited the same trends. Drug releasing biomaterials were also studied with multiple replicates and demonstrated similar release kinetics among replicates, demonstrating reproducibility.
Randomization	N/A
Blinding	N/A

Reporting for specific materials, systems and methods

We require information from authors about some types of materials, experimental systems and methods used in many studies. Here, indicate whether each material, system or method listed is relevant to your study. If you are not sure if a list item applies to your research, read the appropriate section before selecting a response.

Materials & experimental systems

n/a	Involved in the study	
☐	☒	Antibodies
☒	☐	Eukaryotic cell lines
☒	☐	Palaeontology
☐	☒	Animals and other organisms
☒	☐	Human research participants
☒	☐	Clinical data

Methods

n/a	Involved in the study	
☒	☐	ChIP-seq
☒	☐	Flow cytometry
☒	☐	MRI-based neuroimaging

Antibodies

Antibodies used	1. Neurofilament primary antibody (RT97), Dev. Studies Hybridoma Bank at the University of Iowa., Lot No. 9/28/17<br>2. AlexaFluor Donkey-anti-mouse 488 secondary antibody, ThermoFisher, Catalog No. R37114, Lot No. 1915874
Validation	Both antibodies were purchased from commercial sources and the validation information is available on their websites.

Animals and other organisms

Policy information about studies involving animals; ARRIVE guidelines recommended for reporting animal research

Laboratory animals	Sprague Dawley rats, male and female, P2
Wild animals	This study did not involve wild animals.
Field-collected samples	This study did not involve samples collected in the field.
Ethics oversight	All animal work conducted in this study to harvest cells/tissues for in vitro work was approved by the IACUC at Rensselaer Polytechnic Institute.

Note that full information on the approval of the study protocol must also be provided in the manuscript.
